# Longitudinal assessment of post-surgical physical activity in endometrial and ovarian cancer patients

**DOI:** 10.1371/journal.pone.0223791

**Published:** 2019-10-16

**Authors:** Jessica Gorzelitz, Erin S. Costanzo, Ryan J. Spencer, Meredith Rumble, Stephen L. Rose, Lisa Cadmus-Bertram

**Affiliations:** 1 Department of Kinesiology, University of Wisconsin—Madison, Madison, WI, United States of America; 2 Department of Psychiatry, University of Wisconsin—Madison, Madison, WI, United States of America; 3 Department of Obstetrics and Gynecology, Gynecologic Oncology, University of Wisconsin—Madison, Madison, WI, United States of America; Pennington Biomedical Research Center, UNITED STATES

## Abstract

**Objective:**

Physical activity plays a key role in cancer survivorship. The purpose of this investigation was to (a) describe the post-surgical physical activity trajectories of endometrial (n = 65) and ovarian (n = 31) cancer patients and (b) identify clinical and demographic predictors of physical activity over time.

**Methods:**

96 participants wore an Actiwatch accelerometer for three days at each of three time points (one week, one month and four months) after surgical intervention for their endometrial or ovarian cancer diagnosis. Analyses were conducted using linear mixed effects regression modeling in SAS 9.4.

**Results:**

For both tumor types, although physical activity levels increased with time after surgery, even at four months patients were performing only a small fraction of the 150 minutes of recommended weekly moderate to vigorous physical activity. At 1 week, subjects were completing on average 14 minutes/week (SD = 4) of moderate-to-vigorous physical activity, compared to 14 minutes/week (SD = 2) of moderate-to-vigorous physical activity at four months post-surgery (*p* < .05). Better self-rated health was associated with higher physical activity (*p* = 0.02) in endometrial cancer survivors only. BMI, age, surgery type and use of neoadjuvant chemotherapy were not associated with activity over time.

**Conclusions:**

Our findings suggest that physical activity levels are different for those with better self-rated health, but those individuals are still insufficiently active. This study adds new information describing the trajectories and variables that influence physical activity in gynecologic cancer survivors after surgery and highlights the need for health promotion interventions in this population.

## Introduction

Together, endometrial and ovarian cancers comprise 85,470 new diagnoses per year, or 4.9% of all new cancer cases [1]. Endometrial cancer is the fourth most incident cancer among US women with 63,230 estimated new cases in 2018. It is the most common gynecologic malignancy with a 5-year survival rate of 81% [2]. Although less common than endometrial cancer, ovarian cancer generally carries a more severe prognosis and is the fifth most common cause of cancer death in women [3]. Physical activity is associated with better quality of life and may extend survival after diagnosis for both cancers [4–7]. Furthermore, cancer survivors who engage in regular physical activity have better body composition (including higher levels of lean body mass), lower levels of fatigue and depression, and better sleep and physical functioning than their inactive counterparts [8–13].

For these reasons, the American Cancer Society (ACS) has established guidelines for physical activity, which recommend physical activity throughout the survivorship continuum, while noting the need for individualized recommendations for exercise for those undergoing active treatment [14]. Unfortunately, many survivors never achieve and maintain the level of regular physical activity needed to maximize quality of life after their diagnosis [15–18]. Only 48% of endometrial and 19–31% of ovarian cancer survivors report engaging in the recommended amount of aerobic activity [11, 19–23]. Importantly, across populations, self-reported physical activity tends to be over reported [24, 25]. There is a growing focus on device-based measurement such as accelerometry to provide a more accurate picture of physical activity [16]. For example, accelerometer-based estimates from the National Health and Nutrition Examination Survey (NHANES) show that the average post-treatment cancer survivor performs just 16 minutes of daily activity at a moderate or higher intensity, with approximately 8% of cancer survivors meeting physical activity guidelines [26]. Thus, with the methodological limitations of the self-reported measures and the low levels of previously reported physical activity in gynecologic cancer survivors, there is a need for a device-based measure of activity that take into consideration the trajectory of activity after cancer treatment. There are no published data that report device-based measures of physical activity during the post-surgical period in these cancer types.

A cancer diagnosis marks an opportunity when individuals are attuned to their health status and may be particularly amenable to adopting healthy behaviors [27]. In order to maximize that opportunity, we need understand the natural trajectory of physical activity from active treatment through recovery to determine demographic, clinical and psychological characteristics of patients who are at particular risk for sustained inactivity after treatment. Accelerometers are an ideal tool for this purpose because they allow for the capture of even short bursts of movement. The purpose of this study was therefore to determine patterns of physical activity during the first four months following surgery for ovarian and endometrial cancers. Although endometrial and ovarian cancers have different risk factors and treatment trajectories, including both in this investigation allows for comparison of demographic, psychological and clinical factors that may impact physical activity trajectories in the two disease sites. The aims of the study were to (a) identify the trajectory of physical activity over time for both endometrial and ovarian cancer survivors and (b) determine the extent to which key demographic and medical characteristics predicted recovery in physical activity over time. Answering these questions is critical for the development of thoughtful approaches to improve physical activity and quality of life among gynecologic cancer survivors.

## Materials and methods

The University of Wisconsin Madison IRB approved (ID number 2011-0083-CR008) the original longitudinal observational study to determine behavioral and biological predictors of recovery of quality of life after surgery for gynecologic cancer. Informed written consent was collected from all participants.

### Participants

Participants were 96 women (65 endometrial and 31 ovarian cancer survivors) who were enrolled in a larger, IRB-approved (ID number 2011-0083-CR008) longitudinal observational study to determine behavioral and biological predictors of recovery of quality of life after surgery for gynecologic cancer. Participants were recruited between June 2011 and June 2014. Eligible participants were women 18 years of age or older undergoing surgery for suspected endometrial or ovarian cancer at the University of Wisconsin Comprehensive Cancer Center (UWCCC) identified by gynecologic oncology providers (physicians, fellows, or nurses). Participants needed a confirmed diagnosis of endometrial or ovarian cancer prior to study inclusion. Eligible women were invited to participate in the study by research personnel. Patients not able to attend follow-up visits at the UWCCC were excluded from the study. Our sample is representative of the UWCCC’s catchment area with respect to demographics, and the research was conducted at UWCCC and affiliated UW Health Clinics. Informed written consent was collected from all participants. Patient assessments, including self-report measures of quality life, accelerometer evaluation of rest and activity patterns were completed one week, four weeks, and four months after surgery. Detailed methods have been previously described by Rumble, et al. [28]. Participants were included in this analysis if they had valid accelerometer assessments for at least two of the three time points during their post-operative care.

### Demographic and health predictors

Demographic and clinical predictors used in this analysis were selected based on previously published associations with physical activity or other known relevant associations with cancer survival. Participants reported their education, marital status, race, and family income at the first study assessment. Age and body mass index (BMI, kg/m^2^) at the time of study entry were abstracted from the medical record from a recent physical exam. Cancer stage, surgery type, neoadjuvant chemotherapy (completed), and adjuvant therapy (ongoing) were also abstracted from patient medical records under the supervision of a board-certified oncologist (SR). Self-rated health was included as psycho-social construct variable to assess the way patients perceive their health, capacity and ability which we hypothesize influences physical activity. Participants were asked to report their self-rated health using a five-category variable (excellent, very good, good, fair, pool) [29].

### Physical activity measurement

Wrist-worn accelerometers (Actiwatch 64, Mini-Mitter, Bend, OR) were used to measure rest-activity patterns. Participants wore the accelerometer for a three-day period at one week, four weeks, and four months after surgery. Activity data were collected in one-minute epochs (intervals). While not frequently used in population-based research, the Actiwatch has been validated for measuring physical activity [30–32]. Accelerometers were worn 24 hours per day and sleep versus waking time was determined by the validated device assessing if the participant was active, resting or sleeping. This analysis included only those epochs designated as wake time. Data were downloaded using the Actiware software. Raw epoch-by-epoch counts were exported into SAS 9.4 and processed using counts per minute (CPM) cut-points to determine intensity of physical activity for all epochs classified as activity by the Actiwatch. Physical activity intensities were categorized for active wear periods (light intensity: 750–1499 CPM, moderate intensity: 1500–1999, vigorous intensity: >2000 CPM). These cut-points were selected to be consistent with other activity cut-points for other tri-axial accelerometers, similar to the Actiwatch [33]. Participants needed at least two periods of three days of wear to be included. A valid day was defined as greater than 10 hours of wear as is common practice for accelerometry-based physical activity research [34]. The outcome of interest for this analysis was total daily minutes of moderate to vigorous physical activity, standardized to the number of valid days worn per time point. While light physical activity was included in descriptive analysis, the presented analysis primarily focused on moderate to vigorous physical activity as this is the activity intensity in the federal activity guidelines for adults including cancer survivors.

### Statistical analyses

Descriptive statistics for demographic and medical characteristics of the both cancer groups were computed. Body mass index was treated as a continuous variable and cancer stage (I-II vs. III-IV), surgery type (laparoscopic vs. laparotomy), adjuvant therapy use (yes vs. no), and neoadjuvant chemotherapy (yes vs. no) were treated as dichotomous variables. Responses at the first time point were used to predict physical activity over time. These predictors were modeled as follows: age was measured in years, centered at the mean of 59.5 years and was treated as a time-invariant variable since the duration of the study was only four months; BMI was treated as a time-varying covariate; neoadjuvant chemotherapy and surgery type were both indicator variables, treated as time invariant predictors; the single item of self-rated health was a time-invariant covariate measured at the first time point, treated as a five-level categorical variable. Significance was set at alpha = 0.05, but the interpretation of the p-values should be made with caution as the chance of Type II error rate is higher with small sample sizes.

Because endometrial and ovarian cancers have different etiologies and risk factors, each primary disease site had a restricted analysis, as well as a fully saturated model to assess the significance of clinical predictors on the amount of physical activity over time. In univariate association testing, surgery type was not associated with ovarian cancer or physical activity, therefore was not included in the final ovarian cancer model. For endometrial cancer, neoadjuvant therapy was not included in the models because only one participant with endometrial cancer received that treatment, and fewer than 30% of the endometrial patients receiving adjuvant chemotherapy. Analyses were repeated including who did and did not receive brachytherapy and the results were not significantly different. Linear mixed effects (LME) modeling was used with both a random slope and random intercept. When using an LME model, the mean response of total physical activity in minutes (standardized to a week) minutes is modeled as a combination of population characteristics (fixed effect) assumed to be shared by all subjects in the study, and subject-specific effects (random effects) which are unique to everyone. Trajectories of moderate to vigorous physical activity were modeled as functions of time since surgery. Light physical activity was not included in the regression modeling presented, but was included in descriptive analysis. In preliminary model testing, light physical activity was modeled as the outcome variable and the covariates of influence found for light physical activity were not significantly different than for MVPA (data not shown). Assumptions include the introduction of a random subject effect induces correlation among the repeated measure, and random level effects and errors are mutually independent. Additionally, this multilevel model introduces partial pooling effect, where the estimate of an intercept is a weighted average between the unpooled estimate and the completely pooled estimate.

## Results

### Participants

Table 1 provides demographic and medical variables stratified by primary disease site. Women were, on average, 59.5±10.3 years of age with BMI of 36.0±10.6 kg/m^2^; 93.8% were non-Hispanic White. Endometrial vs. ovarian cancer survivors were comparable with respect to all demographic variables. As expected, tumor stage differed, with 84.1% of endometrial cancer survivors diagnosed at Stage I-II versus 15.9% of ovarian patients (*p* < .0001). Endometrial cancer survivors had higher mean BMI of 39.3 kg/m^2^ verses 29.3 kg/m^2^ for ovarian patients (*p* < .0001), which is still on the cusp of obesity for ovarian cancer patients despite a possible weight loss as a result of the cancer diagnosis [35].

**Table 1:** Demographic characteristics for endometrial and ovarian cancer survivors with valid accelerometer data at one week after surgery.

	Overall	Endometrial	Ovarian	*p*-value
	Mean (SD) or n (%)	Mean (SD) or n (%)	Mean (SD) or n (%)	
N	96	65 (67%)	31 (33%)	< .0001
Age in years	59.5 (10.3)	60.3 (9.3)	57.6 (12.1)	.19
Stage I-II (vs. III-IV)	63 (65.6%)	53 (81.5%)	10 (32.2%)	<0.0001
BMI (kg/m^2^)	36.0 (10.6)	39.3 (10.8)	29.3 (6.0)	< .0001
Healthy (BMI <24.9)	15 (15.6%)	6 (9.2%)	9 (29.0%)	< .0001
Overweight (25–29.9)	13 (13.5%)	6 (9.2%)	7 (22.6%)	< .0001
Obese I (30–34.9)	18 (18.8%)	9 (13.9%)	9 (29.0%)	< .0001
Obese II (35 +)	50 (52.1%)	44 (67.7%)	6 (19.4%)	< .0001
Married/partnered	57 (59.4%)	37 (56.9%)	20 (64.5%)	.51
Non-Hispanic White	90 (93.8%)	61 (93.8%)	29 (93.6%)	.66
Education				.29
HS graduate or less	19 (19.8%)	10 (15.4%)	9 (29.0%)	
Trade school/some college	24 (25.0%)	17 (26.2%)	7 (22.6%)	
College graduate	23 (24.0%)	18 (27.6%)	5 (16.1%)	
Post-graduate degree	27 (28.1%)	17 (26.2%)	10 (32.3%)	
Missing	3 (3.1%)	3 (4.6%)	-	
Annual family income				.65
≤$25,000	19 (19.8%)	12 (18.5%)	7 (22.6%)	
$25,001 –$55,000	25 (26.0%)	18 (27.7%)	7 (22.6%)	
$55,001 –$85,000	24 (25.0%)	15 (23.1%)	9 (29.0%)	
>$85,000	22 (22.9%)	17 (26.2%)	5 (16.1%)	
Missing	6 (6.3%)	3 (4.6%)	3 (9.7%)	
Received neoadjuvant chemotherapy	9 (9.4%)	1 (1.5%)	8 (25.8%)	<0.0001
Received adjuvant therapy	57 (59.4%)	30 (52.6%)	27 (87.1%)	<0.0001
Laparoscopy (vs. open surgery)	N/A	43 (66.2%)	N/A	
Self-rated health				.80
Excellent	10 (10.4%)	8 (12.3%)	2 (6.5%)	
Very good	30 (31.3%)	21 (32.3%)	9 (29.0%)	
Good	30 (31.3%)	18 (27.7%)	12 (38.7%)	
Fair	15 (15.5%)	10 (15.4%)	5 (16.1%)	
Poor	2 (2.1%)	1 (1.5%)	1 (3.2%)	
Missing	9 (9.4%)	7 (10.8%)	2 (6.5%)	

### Physical activity

**Fig 1:**
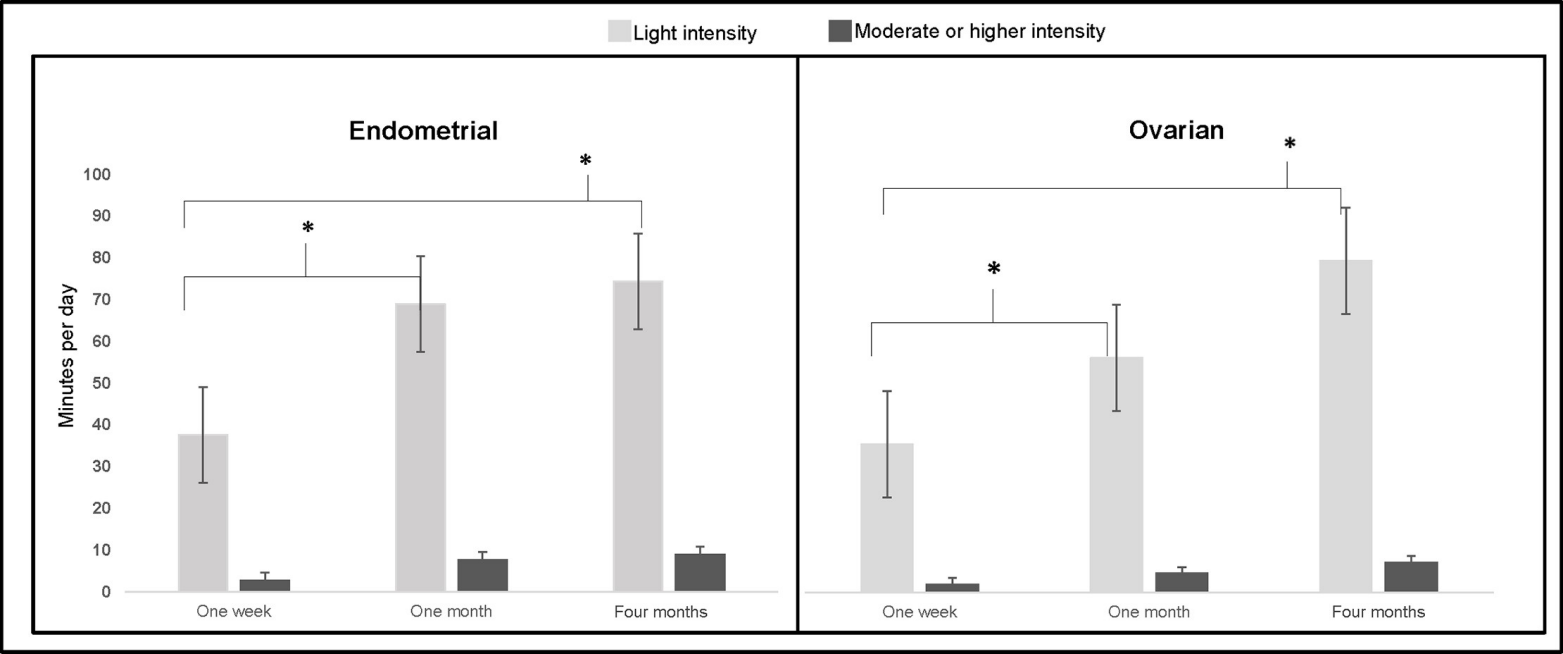
Standardized daily minutes of activity stratified by intensity and primary disease site. Asterisks denote statistical significance at the *p* < .05 level.

Most of the activity accumulated at each time point was performed at a light intensity, with a very small proportion of total physical activity performed at an intensity of moderate or higher (Fig 1). Endometrial cancer survivors performed 114 minutes/week (SD = 111) of light-intensity activity one week after surgery, 193 minutes/week (SD = 148) one month after surgery, and 232 minutes/week (SD = 149) four months after surgery. These changes were statistically different from one week to four months (*p* < .0001) but not different between one month and four months after surgery (*p* = .06). Similarly, ovarian cancer survivors performed 108 minutes/week (SD = 113) of light intensity activity at one week, 171 minutes/week (SD = 141) at one month, and 242 (141) at four months which were also statistically different between one week and four months (*p* = .02) but not different between one month and four months after surgery (*p* = .16). The duration of activity performed at a moderate or higher intensity was minimal. At one week post-surgery, participants engaged in an average of 14 (SD = 4) minutes of weekly activity of at least moderate intensity. At one month after surgery, there was an increase of to 49 minutes/week (SD = 14), and at four months post-surgery the weekly amount of moderate-plus activity was at 14 minutes (SD = 2). Data are presented as weekly estimates to map on to the ACS guidelines, which was gathered from a three-day wear period and standardized to a seven-day estimate. Fig 1 presents these data as daily estimates to supplement the weekly estimates. These differences were statistically significant from one week to one month (*p* = .002) post-surgery and were also significant from one week to four months (*p* < .0001) post-surgery. However, MVPA between one-month and four months after surgery was not statistically significant. No participant at any time point met the American Cancer Society recommendation of at least 150 minutes/week of moderate intensity activity.

### Predictors of physical activity (mixed effects modeling)

Table 2 provides unadjusted estimates of total daily moderate-to-vigorous physical activity and each clinical or demographic characteristic included in the final model. Neither disease stage nor use of adjuvant therapy were associated with physical activity when tested for inclusion in models; therefore, these variables were therefore excluded from the final models. A robustness check was conducted by re-running the final models with these variables included and they were not significantly associated with the outcome for either disease site (results not shown).

**Table 2:** Cancer-specific unadjusted (top) and mixed effects (bottom) models for predictors of moderate-to-vigorous physical activity over four months.

**UNADJUSTED MODELS**		
	**Endometrial**	**Ovarian**
	**Beta (95% CI)**	**Beta (95% CI)**
Time from surgery	1.0 (0.3, 1.8) *	0.8 (-0.0, 1.6) *
Age	-0.3 (-1.0, 0.5)	-0.3 (-0.7, 0.1)
BMI	-0.5 (-1.0, 0.1)	0.0 (-0.8, 0.8)
Laproscopy vs. open surgery^a^	4.5 (-10.1, 19.2)	N/A
Neoadjuvant chemotherapy^b^	N/A	-7 (-18, 3.8)
Self-rated health^c^	-11.3 (-17.7, -4.8) *	-5.3 (-10.8, 0.2) *
**ADJUSTED MIXED EFFECTS MODELS**		
	**Endometrial**	**Ovarian**
	**Beta (95% CI)**	**Beta (95% CI)**
Intercept	43.7 (15.8, 71.6)*	22.0 (-7.0, 51.0)
Time from surgery	0.9 (0.2, 1.6)*	0.8 (0.0, 1.7)*
Age	-0.4 (1.0, 0.3)	-0.2 (-0.7, 0.3)
BMI	-0.3 (-0.9, 0.3)	0.1 (-0.8, 0.8)
Laproscopy vs. open surgery^a^	-0.1 (-12.6, 12.5)	N/A
Neoadjuvant chemotherapy^b^	N/A	-5.2 (-18.4, 8.0)
Self-rated health^c^	-7.3 (-13.2, -1.4)	-5.0 (-10.7, 0.7)

* indicates statistical significance (*p* < .05)

^a^ In univariate testing, surgery type was not associated with ovarian cancer or physical activity, therefore was not included in the final ovarian cancer model.

^b^ Few women with endometrial cancer received neoadjuvant chemotherapy therefore it was only included in the ovarian cancer models.

^c^ Self-rated health is scored from 1–5, with excellent health (response = 1) as the reference; therefore, poor health is the worst category and is scored as 5 which produces the negative beta estimate.

For both primary disease sites, time from surgery and self-rated health scores independently predicted total moderate-to-vigorous physical activity, with longer time since surgery and better self-rated health independently associated with more activity. Without adjusting for other factors, moving up one category in self-rated health (e.g., from rating one’s health as ‘very good’ to ‘excellent’) was associated with an increase of 11 minutes/week of moderate-to-vigorous physical activity for endometrial cancer survivors (*p* = .02) and an increase of 5 minutes/week for ovarian cancer survivors (*p* = .03). Each additional week in time since surgery was independently associated with an increase of one minute/week of moderate-to-vigorous physical activity for both endometrial (*p* = .02) and ovarian (*p* = .05) cancer survivors.

Table 2 presents the results from separate models for endometrial and ovarian cancer patients with a focus on clinical factors predicted to differ between these two survivor groups. When adjusted for all predictors, time since surgery was positively associated with moderate to vigorous physical activity for endometrial patients (*p* = .02) and ovarian patients (*p* = .05). Self-rated health was significantly associated with increased moderate-to-vigorous physical activity over time for endometrial patients (*p* = .02) but not for ovarian cancer patients. To assess appropriate model selection, both residual variance and BIC (Bayesian Information Criterion) decreased comparing the univariate associations with the full model. For the final models presented, plot diagnostics revealed improved residuals compared to the unadjusted univariate models presented in Table 2.

Table 3 is a fully saturated model with interaction terms for primary disease site and predictors of interest. A fully saturated model is presented due to limited sample size for the ovarian cancer patients compared to the endometrial cancer patients and serves as a robustness check to determine if the associations found in Table 2 persisted. *Main effects*: Main effects persisted for time since surgery and for self-rated health. Time since surgery remained positively associated with moderate to vigorous physical activity for both primary disease sites (*p* = .0015). Higher self-rated health was still associated with higher moderate to vigorous physical activity over time for both primary disease sites (*p* = .0007). *Interaction effects*: No interaction effects tested contributed significantly to the model. No group interaction was found between primary disease site and age, and primary disease site and BMI in any models presented. Group-level interaction terms between primary disease site and self-rated health, between primary disease sites and neoadjuvant chemotherapy, and primary disease site and laparotomy were all not statistically significant showing a lack of evidence for heterogeneity between ovarian and endometrial cancer patients and predictors of neoadjuvant chemotherapy, laparotomy and self-rated health.

**Table 3:** Mixed effects model for predictors of physical activity among endometrial and ovarian cancer survivors^a^.

Effect	Estimate (SE)	*P* value
Intercept	47.8* (13.0)	.0004
Time since surgery	0.8* (0.3)	.0015
Age^b^	-0.4 (0.2)	.10
BMI	-0.4 (0.3)	.19
Self-rated health^c^	-9.6* (2.8)	.0007
Primary disease site^d^	-14.0 (14.7)	.35
Laparoscopic surgery^e^	5.2 (5.9)	.38
Use of neoadjuvant chemotherapy^f^	31.4 (27.8)	.26
Primary disease site × Self-rated health	3.5 (4.8)	.47
Primary disease site × Neoadjuvant chemotherapy	-33.6 (29.4)	.26
Primary disease site × Laparoscopy	-2.4 (14.4)	.87

* indicates statistical significance (*p* < .05)

^a^ Cancer-specific saturated models are not presented because they were not significantly different than those presented in Table 3

^b^ Centered at the mean of 59.5 for ease of interpretation

^c^ Self-rated health is scored from 1–5, with excellent health (response = 1) as the reference; therefore, poor health is the worst category and is scored as 5 which produces the negative beta estimate

^d^ Endometrial cancer is reference, and ovarian cancer is indicator category = 1

^e^ Indicator only present for endometrial cancer, laparotomy is the reference category with laparoscopy as the category = 1

^f^ Indicator only present for ovarian cancer, use of neoadjuvant chemotherapy is category = 1

## Discussion

This study used accelerometers to measure trajectories of physical activity change during the first 4 months after surgery for endometrial or ovarian cancer. The most important finding is that physical activity levels remained extremely low even four months after surgery. This is especially notable given the relatively high income and education level of our sample, as these factors are reliably associated with higher physical activity [36, 37]. Therefore, activity levels in a more representative group of endometrial and ovarian cancer survivors would likely be even lower. Even in our well-educated sample, no participant met the American Cancer Society’s guideline within four months of surgery, although it bears mentioning that the ACS guidelines were based on evidenced derived from self-reported estimates of activity. Since self-report leads to over-estimations of physical activity, measuring activity via devices could lead to misclassification of those who are sufficiently active.

It should be noted that the American Cancer Society’s guidelines are intended broadly to communicate that cancer survivors should strive, as appropriate, to attain the same level of physical activity that is recommended for healthy adults. The population in this analysis was undergoing or recovering from active treatment, thus it is not expected that they would achieve the full recommendation of 150 minutes of moderate to vigorous physical activity per week within four months of surgery. However, our results demonstrating negligible increase in moderate-to-vigorous physical activity by four months post-surgery suggest that survivors are not on track to recover to a volume of physical activity that is even close to the recommended level.

The results show an overall increasing trend in light physical activity from one week to four months post-surgery, indicating that survivors are indeed recovering and increasing their overall physical activity. However, the intensity of the activity is important to consider with many health benefits conferred at intensity levels of moderate or higher based on a self-reported intensity. Perhaps while the overall activity is recovering, there is not enough emphasis on moderate to vigorous physical activity intensity especially during the early recovery period. Recent studies with endometrial cancer survivors have shown that interventions that included wrist-worn Fitbits, group coaching sessions, and telephone calls are efficacious in increasing physical activity [38, 39]. However, these interventions focus on those diagnosed within the last five years who may not be exchangeable with those with a proximal surgery from which to recover.

Contrary to expectations, clinical predictors such as invasiveness of surgery, neoadjuvant chemotherapy, use of adjuvant therapy, and cancer site (ovarian vs. endometrial cancer) were not associated with physical activity over time. This may be due to a floor effect given the extremely low levels of physical activity in the sample; these treatments may have affected sufficiently active individuals but there is little room for change among inactive individuals. However, better self-rated health was associated with higher levels of moderate-to-vigorous physical activity, which is consistent with other published literature [40–42]. Self-rated health is a simple metric that may help identify those individuals who would benefit most from lifestyle interventions.

This study extends the field of physical activity and cancer survivorship by presenting device-measured, longitudinal data collected during the post-surgical period. Additional strengths include a low risk of reactivity (i.e., women being more active because they know their activity is being measured) because the parent study focused primarily on sleep and quality of life rather than physical activity. Other strengths include the prospective, longitudinal design and the inclusion of two disease sites.

### Study limitations

Study limitations include lack of a pre-surgical assessment of physical activity levels (as previous experience with physical activity is a predictor of future behavior), limited generalizability given the relatively homogeneous demographic characteristics with respect to race and ethnicity, and a small sample size for each disease site. The absolute changes observed between time points appear meaningful, highlighting the importance of replicating the findings with a larger sample. Specific surgical restrictions following the more invasive surgeries are unknown and may have affected physical activity levels. However, this limitation is relatively minor with majority (66%) of the sample undergoing minimally invasive surgery. Finally, the Actiwatch is frequently used by sleep researchers and is not used as commonly as the ActiGraph for physical activity assessment.

## Conclusions

These findings highlight the need to help women recovering from gynecologic cancer treatment increase physical activity. Future work might capitalize on a new cancer diagnosis by evaluating the feasibility and tolerability of an intervention to increase activity during the post-surgical recovery period, especially for women with endometrial cancer [32]. Additionally, given that regular physical fitness is associated with better surgical outcomes especially in a pre-surgical exercise intervention setting [33], a special emphasis should be placed on engaging the physician/oncologist in the behavior change process, as previous literature has shown that the physician recommendations towards physical activity can be effective [34].
